# Leveraging anatomical constraints with uncertainty for pneumothorax segmentation

**DOI:** 10.1002/hcs2.119

**Published:** 2024-12-15

**Authors:** Han Yuan, Chuan Hong, Nguyen Tuan Anh Tran, Xinxing Xu, Nan Liu

**Affiliations:** ^1^ Centre for Quantitative Medicine, Duke‐NUS Medical School Singapore; ^2^ Department of Biostatistics and Bioinformatics Duke University Durham North Carolina USA; ^3^ Department of Diagnostic Radiology Singapore General Hospital Singapore; ^4^ Institute of High Performance Computing, Agency for Science, Technology and Research Singapore; ^5^ Programme in Health Services and Systems Research, Duke‐NUS Medical School Singapore; ^6^ Institute of Data Science National University of Singapore Singapore

**Keywords:** constrained optimization, deep transfer learning, diagnostic radiology, pneumothorax detection, semantic segmentation

## Abstract

**Background:**

Pneumothorax is a medical emergency caused by the abnormal accumulation of air in the pleural space—the potential space between the lungs and chest wall. On 2D chest radiographs, pneumothorax occurs within the thoracic cavity and outside of the mediastinum, and we refer to this area as “lung + space.” While deep learning (DL) has increasingly been utilized to segment pneumothorax lesions in chest radiographs, many existing DL models employ an end‐to‐end approach. These models directly map chest radiographs to clinician‐annotated lesion areas, often neglecting the vital domain knowledge that pneumothorax is inherently location‐sensitive.

**Methods:**

We propose a novel approach that incorporates the lung + space as a constraint during DL model training for pneumothorax segmentation on 2D chest radiographs. To circumvent the need for additional annotations and to prevent potential label leakage on the target task, our method utilizes external datasets and an auxiliary task of lung segmentation. This approach generates a specific constraint of lung + space for each chest radiograph. Furthermore, we have incorporated a discriminator to eliminate unreliable constraints caused by the domain shift between the auxiliary and target datasets.

**Results:**

Our results demonstrated considerable improvements, with average performance gains of 4.6%, 3.6%, and 3.3% regarding intersection over union, dice similarity coefficient, and Hausdorff distance. These results were consistent across six baseline models built on three architectures (U‐Net, LinkNet, or PSPNet) and two backbones (VGG‐11 or MobileOne‐S0). We further conducted an ablation study to evaluate the contribution of each component in the proposed method and undertook several robustness studies on hyper‐parameter selection to validate the stability of our method.

**Conclusions:**

The integration of domain knowledge in DL models for medical applications has often been underemphasized. Our research underscores the significance of incorporating medical domain knowledge about the location‐specific nature of pneumothorax to enhance DL‐based lesion segmentation and further bolster clinicians' trust in DL tools. Beyond pneumothorax, our approach is promising for other thoracic conditions that possess location‐relevant characteristics.

## Graphical abstract

We proposed a novel approach that incorporates the lung + space as a constraint during deep learning model training for pneumothorax segmentation on 2D chest radiographs. We utilized external datasets and an auxiliary task of lung segmentation to generate a specific constraint of lung + space for each chest radiograph, circumventing the need for additional annotations. We incorporated a discriminator to eliminate unreliable constraints caused by the domain shift between the auxiliary and target datasets. Our results demonstrated consistent improvements across six baseline models built on three segmentation architectures and two convolutional neural networks backbones.

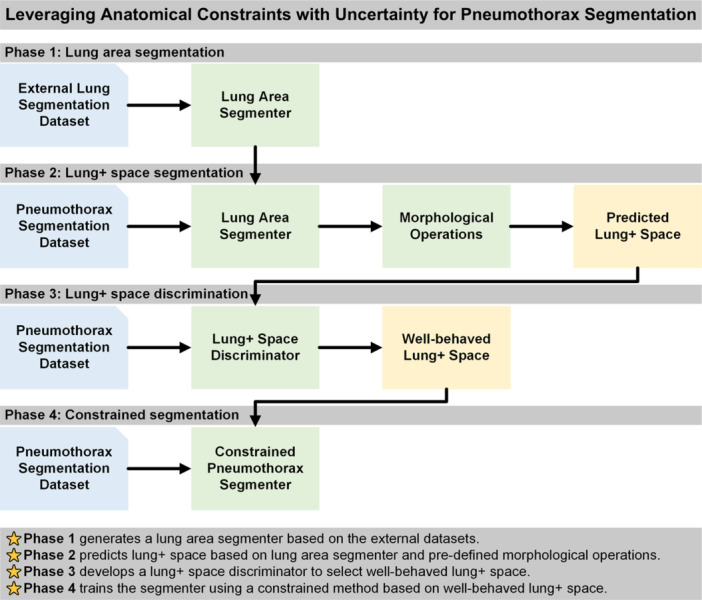

**Abbreviations**
ACR: American College of Radiology
AUROC: area under the receiver operating characteristic curve
DL: deep learning
DSC: dice similarity coefficient
HD: Hausdorff distance
IoU: intersection over union
JSRT: Japanese Society of Radiological Technology
MC: Montgomery County data set
ML: machine learning
NPV: negative predictive value
PPV: positive predictive value
SGD: stochastic gradient descent
SIIM: Society for Imaging Informatics in Medicine

## 1 BACKGROUND

### 1.1 Motivation

Pneumothorax is a medical emergency caused by the abnormal accumulation of air in the pleural space, which is the potential space between the lungs and chest wall [1, 2]. The pleural space is non‐existent in healthy controls, whereas it can occupy significant portions of the thoracic cavity in patients with a large pneumothorax. Chest radiographs serve as the primary diagnostic tool for pneumothorax, aiding in identifying its location and estimating its size [3, 4]. Traditionally, radiologists report chest radiographs based on their domain knowledge and past experience [5, 6]. In recent years, with the advent of machine learning (ML), especially deep learning (DL), there has been a shift towards automated detection and segmentation of pneumothorax from chest radiographs, achieving promising performance when paired with high‐quality annotations [7, 8]. Conventional ML methods focus on identifying contrast regions from chest radiographs [9, 10] and performing morphological operations such as atlas guidance [11], contour deformation [10], and inhomogeneity correction [8] to delineate lesion segmentation. Compared with conventional ML methods involving multiple stages that allow for the incorporation of domain knowledge [12, 13], DL methods typically follow the end‐to‐end paradigm [14] to capture intricate patterns and contextual information in chest radiographs and offer more fine‐grained lesion segmentation. However, end‐to‐end approaches often neglect the fact that pneumothorax is inherently location‐sensitive, predominantly manifesting within the pleural space [15, 16]. Our approach aims to leverage the medical domain knowledge of disease occurrence into DL‐based pneumothorax segmentation, emphasizing the significance of the location‐specific nature of the disease.

### 1.2 Related work

#### 1.2.1 DL‐aided diagnosis for pneumothorax

DL‐aided tools have emerged as promising solutions for diagnosing pneumothorax from chest radiographs [17]. Numerous studies have reported accurate classification using various DL models [18], such as AlexNet [19, 20], VGG [19, 21], Inception [19, 22], EfficientNet [23, 24], Network in Network [25], ResNet [26, 27], DenseNet [19, 28], AlbuNet [29, 30], Spatial Transformer [31, 32], and others. While most studies employ the conventional end‐to‐end approach, Chen et al. designed a two‐stage model for pneumothorax classification [33], combining an object detection model YOLO [34] and classification techniques ResNet [27] and DenseNet [28] to distinguish pneumothorax patients and healthy individuals based on the cropped lung field.

Recent research has expanded beyond merely image‐level labeling, emphasizing pixel‐level lesion area delineation. This not only reduces radiologists' workload but also fosters greater trust in automated systems [35, 36]. Like the binary classification of pneumothorax, DL‐based techniques are the state‐of‐the‐art choices for pneumothorax area segmentation [37]. The techniques used mainly comprise the architectures of U‐Net [38, 39], DeepLab [40, 41], Mask R‐CNN [40, 42], with the backbones of ResNet [39, 43], SE‐ResNext [40, 43], DenseNet [15, 43], EfficientNet [40]. Apart from tailoring models on specific datasets, commercial systems, such as DEEP: CHEST‐XR‐03, have also been developed and clinically validated for pneumothorax segmentation [44].

#### 1.2.2 Domain knowledge in chest radiograph analysis

While most of the models mentioned above employ an end‐to‐end approach, mapping input chest radiographs directly to target disease labels or lesion annotations, the integration of domain knowledge in medical image analysis is pivotal [45, 46]. Incorporating such knowledge not only enhances model interpretability but also boosts model performance [47]. In chest radiograph analysis, disease localization serves as invaluable prior knowledge [48]. For example, Li et al. identified specific anatomical regions within the lung zone, highlighting their diagnostic relevance in detecting conditions of cardiomegaly and pleural effusion [49]. Similarly, Crosby et al. focused on the upper third of chest radiographs and demonstrated that a VGG‐based classifier trained barely on the sub‐region yielded good performance in the task of distinguishing pneumothorax [50]. Recently, Jung et al. proposed to extract the domain knowledge of thoracic disease occurrence from the class activation map of a thoracic disease classifier. The extracted knowledge was then used for precise thoracic disease localization [51]. Such methods typically utilize masks at various stages, from input to output, ensuring the model's attention is directed towards areas with a higher likelihood of disease presence. More recently, Bateson et al. introduced an innovative method that incorporates prior knowledge about organ size into the model training [52, 53]. By penalizing deviations from this domain knowledge, the model is guided to align more closely with clinical insights. Notably, in tasks of spine and heart segmentation, their method achieved considerably better performance than conventional methods.

### 1.3 Contribution

According to clinical knowledge [1, 2], pneumothorax is an abnormal gas collection in the pleural space, which is a potential space between the lungs and chest wall. On a 2D projection of a chest radiograph, pneumothorax localizes inside the thoracic cavity and outside of the mediastinum. We refer to this area as “lung + space,” which includes the lungs and the pleural space on a chest radiograph. We propose to incorporate the location information into pneumothorax segmentation. Inspired by [54, 55], we employed occurrence information as a guiding constraint in the segmentation training. To circumvent the need for additional domain knowledge annotation, we proposed a four‐phase pipeline to determine the disease occurrence area. Leveraging both external open‐access datasets and existing annotations, our evaluations, conducted on a widely‐recognized pneumothorax segmentation data set, revealed that our constrained training approach consistently outperformed traditional methods across a range of architectures and backbones. Moreover, to validate the efficacy of our approach, we have conducted an ablation study and two robustness experiments. These studies underscored the effectiveness of individual components within our design and affirmed the overall stability of our proposed method. We hope the proposed method provides a useful framework for embedding domain knowledge into the diagnosis of other thoracic conditions that possess location‐relevant characteristics.

## 2 METHODS

To harness the power of domain knowledge in improving DL efficacy in lesion segmentation, we propose a four‐phase pipeline that incorporates disease occurrence knowledge into the training stage of the pneumothorax segmenter. In this section, we first provide an overview of the proposed pipeline. We then detail the generation process of anatomical constraints. Finally, we elaborate on the constrained training strategy for lesion segmentation.

### 2.1 Overview of the proposed pipeline

**Figure 1:**
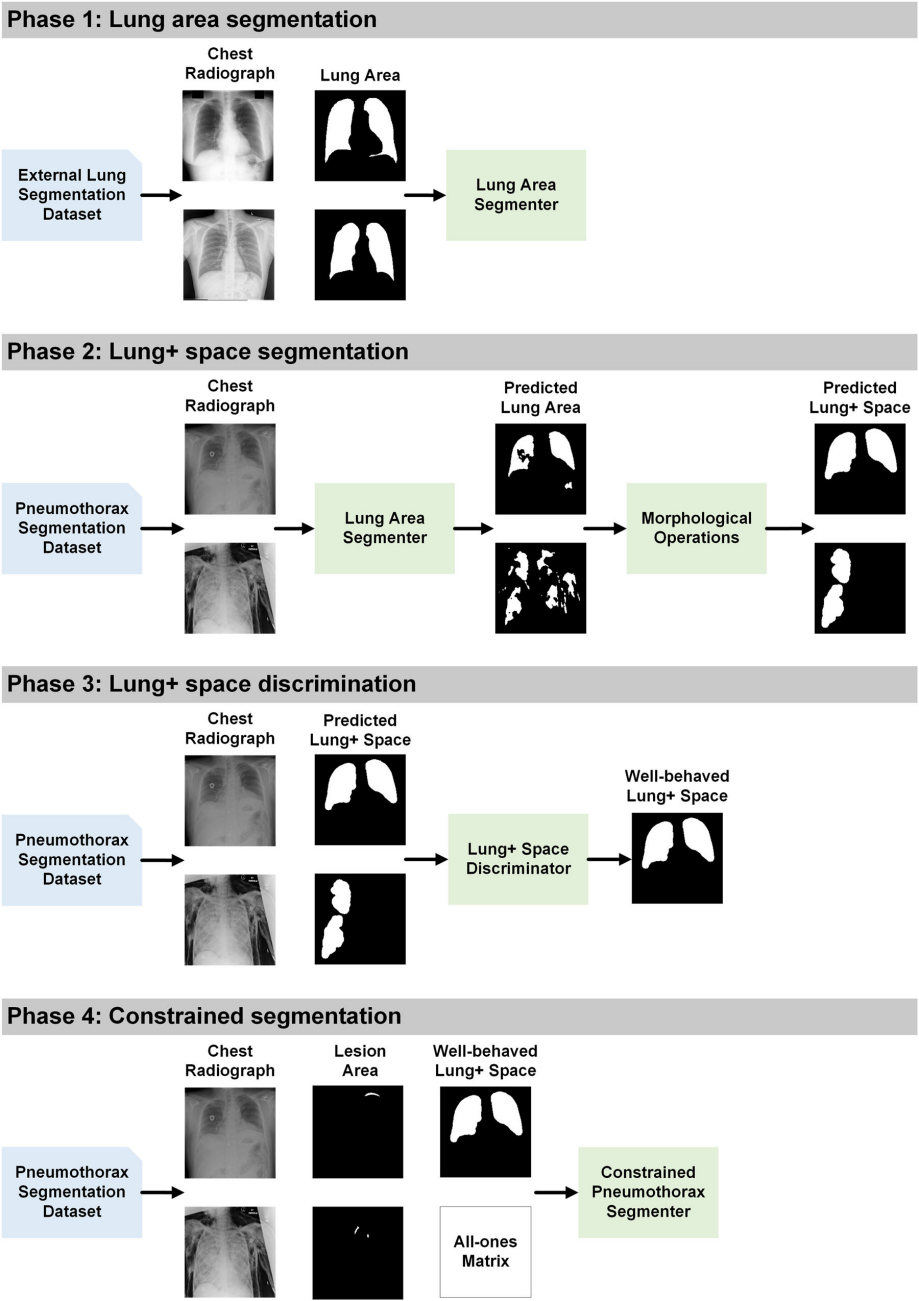
Schematic diagram of the proposed constrained segmentation framework.

As outlined in Figure 1, the proposed pipeline contains four indispensable phases to obtain sample‐specific lung + space, select well‐behaved lung + space, and implement constrained training of pneumothorax segmenter. In Phase 1, we develop an auxiliary lung segmenter using three public lung segmentation datasets, including Japanese Society of Radiological Technology data set (JSRT) [56], Shenzhen data set (Shenzhen) [57], and Montgomery County data set (MC) [57]. This segmenter is then integrated with morphological operations, including connected component cutoff, closing, and dilation to derive a lung + space segmenter in Phase 2. This refined segmenter is subsequently deployed on the target data set of pneumothorax segmentation to predict lung + space. In Phase 3, we introduce a lung + space discriminator, crafted using the training and validation data set for the pneumothorax segmentation, filtering out inaccurately predicted lung + spaces from Phase 2, ensuring only high‐quality constraints are retained. In Phase 4, with the selected lung + space from Phase 3, we proceed to train the pneumothorax segmenter using a constrained approach. To detail the four phases, the remaining parts are organized as follows: First, we provide in Section 2.2 the design details of Phases 1, 2, and 3 for generating the well‐behaved anatomical constraints for pneumothorax, including three coherent modules of lung area segmenter, lung + space segmenter, and lung + space discriminator. Second, we illustrate the formulation of a standard lesion segmenter and outline the proposed constrained segmenter of Phase 4 in Section 2.3.

### 2.2 Learning the anatomical constraints

The primary challenge in our proposed method lies in the derivation of well‐behaved constraints. From the domain knowledge, we understand that pneumothorax occurs in the lung + space and the lung + space can be used as a constraint in segmenter training. The previous work generally requires constraint‐relevant annotation on the target data set, which incurs additional expenses and lacks adaptability when extended to other datasets [54, 58]. To tackle this problem, we exploit three external open‐access datasets and an auxiliary task of lung segmentation to generate the lung + space as our anatomical constraints. Specifically, we first generate a lung area segmenter based on the external datasets. Then, we integrate several post‐processing techniques into the lung area segmenter to achieve a lung + space segmenter and deploy it on the target data set. Finally, considering the inherent noise in the constraints generated from the external source‐based model, a lung + space discriminator is developed to filter out the noisy constraints while retaining the informative ones for the constrained training of the pneumothorax segmenter.

#### 2.2.1 Phase 1: Lung area segmentation

The foundation for our anatomical constraint learning is the lung area segmenter trained on external datasets. We utilize three publicly available chest radiograph datasets with lung area annotations and develop the lung area segmenter following the standard area segmentation [56, 57]. An intuitive idea is to transfer the trained lung area segmenter to the target data set, obtain the lung area, and use them as the constraints. However, according to domain knowledge, pneumothorax is an abnormal gas (lucent area) in the pleural space, which is localized in the lung + space on 2D chest radiographs. Additionally, the directly deployed lung segmenter suffers from a domain shift between the source data set and the target data set [59, 60]. Consequently, certain portions of the generated lung area may exhibit detrimental noise to the downstream pneumothorax segmentation. To tackle the first challenge, we integrate several morphological operations into the fundamental lung area segmenter to derive the lung + space segmenter. To address the second issue, we design a lung + space discriminator to filter out the noisy constraints of lung + space and retain the informative ones.

#### 2.2.2 Phase 2: Lung + space segmentation

Based on the auxiliary lung segmenter, we implement three post‐processing steps, including the largest connected component cutoff, morphological closing, and morphological dilation to develop a lung + space segmenter. The initial operation, the largest connected component cutoff, is employed to mitigate the impact of small islands, a common issue in medical image segmentation [61, 62]. By retaining the top two largest areas, we effectively extract the two frontal lung regions from the noisy segmentation output. Subsequently, morphological closing is applied to fill small holes inside the lung area, another prevalent concern in medical image segmentation [63, 64]. Lastly, morphological dilation is employed to extend the lung area, encompassing the side pleural space between the lung boundary and the chest wall [65, 66]. By combining these post‐processing operations with the lung area segmenter, we have effectively crafted the lung + space segmenter which generates the candidate constraints of lung + space for each chest radiograph in the target data set of pneumothorax segmentation.

#### 2.2.3 Phase 3: Lung + space discrimination

Utilizing the lung + space segmenter, each chest radiograph $I_{i}$ in the target task has been enriched with a constraint $C_{i}$ Which holds an identical shape as $I_{i}$. In a constraint $C_{i}$, pixels located within the lung + space are assigned a value of 1, while those outside the lung + space are marked as 0. Nevertheless, due to the problem of domain shift [52, 67], some of the constraints still present noise issues. If all constraints are unquestioningly incorporated in model training, the improvements achieved by well‐behaved constraints can potentially be negated by the detrimental constraints. To address this challenge, a lung + space discriminator is developed to differentiate whether a predicted lung + space contains significant noise.

The constraint discrimination constitutes a binary classification task. For training the discriminator, Equation (1) derives binary labels by calculating the coverage rate $R_{i}$ of pneumothorax annotation $S_{i}$ in relation to the lung + space constraint $C_{i}$. If $R_{i}$ exceeds τ, the binary label $B_{i}$ is designated as 1; otherwise, it is set to 0.

(1) $$R_{i}=\frac{\left|C_{i}\cap S_{i}\right|}{\left|S_{i}\right|}$$

Regarding the discriminator input, we followed a previous study [68] to fuse the original chest radiograph $I_{i}$, the constraint $C_{i}$, and the masked chest radiograph ${I_{i}\times C}_{i}$ on the channel level. The model output is configured to yield a single value, predicting the probability of including the constraint in the training phase. With the inputs, output targets, and the binary classifier, we proceed with the standard image classification using cross‐entropy loss [65]. After the binary classification, certain chest radiographs are augmented with the constraints of lung + space. For those samples whose constraints are excluded, we supplement them with all‐one matrixes to align the data format and nullify the constraint effect. Finally, we obtain the anatomical constraints of lung + space for the downstream training of the constrained segmenter. It is noteworthy that the classification is performed exclusively on the training and validation sets of the target datasets, ensuring that the test set is unseen and there is no information leakage.

### 2.3 Constrained segmentation

We first present a standard formulation of image segmenter training without any constraints, which serves as the baseline for our study. Subsequently, we elaborate on the constrained training approach, which includes an additional penalty term aimed at satisfying the imposed constraints.

In a typical training stage of a single disease segmenter, we consider a data set D consisting of N input images $I_{i},\;i=1,\ldots ,\;N$ and their respective lesion segmentations $S_{i},\;i=1,\ldots ,\;N$. Then the data set D is randomly split into training set $D_{\mathrm{train}}$, validation set $D_{\mathrm{valid}}$, and test set $D_{\mathrm{test}}$ with $N_{\mathrm{train}}$, $N_{\mathrm{valid}}$, and $N_{\mathrm{test}}$ samples, respectively. After that, a model Y with parameter θ is trained on $D_{\mathrm{train}}$, by minimizing the overall loss L averaging sample‐wise loss l such as Dice or cross‐entropy [69] between the model output $Y(I_{i},\theta )$ and the ground‐truth mask $S_{i}$.

To avoid the overfitting of Y, the optimization of θ is early‐stopped upon reaching the loss plateau on $D_{\mathrm{valid}}$. However, the loss function defined by Equation (2) overlooks the domain knowledge and trains the lesion segmenter through an end‐to‐end approach to map $I_{i}$ to $S_{i}$. In clinical medicine, some diseases highly occur in certain regions, which receive more attention than other areas in the diagnostic process [65, 66]. Therefore, the prior knowledge of the disease occurrence area potentially contributes to the training process of the disease segmenter.

(2) $$L_{D_{\mathrm{train}}}=\frac{1}{N_{\mathrm{train}}}\sum_{i=1}^{N_{\mathrm{train}}}l\left(Y\left(I_{i},\theta \right),S_{i}\right).$$

#### 2.3.1 Phase 4: Constrained lesion area segmentation

In our proposed formulation, the disease occurrence area is introduced as a constraint in the loss function. To guide the model's focus on the disease occurrence area, the adjusted loss function defined by Equation (3) penalizes the model if the constraint is violated. Specifically, the loss function in Equation (2) will be supplemented with a novel penalty term P comparing the model output $Y(I_{i},\theta )$ with a sample‐specific constraint $C_{i}$:

(3) $$L_{D_{\mathrm{train}}}=\frac{1}{N_{\mathrm{train}}}\sum_{i=1}^{N_{\mathrm{train}}}(l\left(Y\left(I_{i},\theta \right),S_{i}\right)+\lambda \times P(Y\left(I_{i},\theta \right),C_{i})),$$

where $l\left(Y\left(I_{i},\theta \right),S_{i}\right)$ stands for the classic loss function for image segmentation in Equation (2), λ is a positive hyper‐parameter fine‐tuned on $D_{\mathrm{valid}}$, and Equation (4) denotes the proposed penalty term $P(Y\left(I_{i},\theta \right),C_{i})$:

(4) $$P\left(Y\left(I_{i},\theta \right),C_{i}\right)=1-\frac{\left|Y\left(I_{i},\theta \right)\cap C_{i}\right|}{\left|Y\left(I_{i},\theta \right)\right|}$$

with $\left|Y\left(I_{i},\theta \right)\cap C_{i}\right|$ standing for the intersection area size between $Y\left(I_{i},\theta \right)$ and $C_{i}$, and $\left|Y\left(I_{i},\theta \right)\right|$ representing the size of $Y\left(I_{i},\theta \right)$. Clearly, when an outputted disease segmentation crosses the constraint boundary, the penalty function is positive while a satisfied segmentation within the constraint area corresponds to a null penalty. By adding such a differentiable term in the loss function, we address the difficulty of conventional convex‐optimization of neural networks for the constraint satisfaction [70, 71].

### 2.4 Experimental settings

#### 2.4.1 Datasets

Our proposed formulation was based on three external datasets of lung segmentation and a target data set of pneumothorax segmentation. Table 1 gives an overview of the used datasets and their purposes in our experiments. The lung segmentation datasets were used to develop the auxiliary lung segmenter, the foundation for the lung + space segmenter. The pneumothorax data set was our target data set to build the lung + space discriminator and compare the pneumothorax segmenter trained by the baseline or the constrained loss function.

**Table 1:** An overview of the used data set, abbreviation, and function.

Data set name	Abbreviation	Purpose
Japanese Society of Radiological Technology data set [56]	JSRT	Lung segmentation Lung + space segmentation
Shenzhen data set [57]	Shenzhen	
Montgomery County data set [57]	MC	
Society for Imaging Informatics in Medicine‐American College of Radiology Pneumothorax Segmentation data set [72]	SIIM‐ACR	Lung + space discrimination
		Pneumothorax segmentation

##### Lung segmentation data set

We developed the lung segmenter using JSRT, Shenzhen, and MC datasets. The JSRT data set was collected by the Japan Radiological Society and it includes 247 chest radiographs with a resolution of 2048 × 2048. The original data set contained no ground‐truth lung area annotation, which was supplemented by another team [73]. The MC data set was gathered by Montgomery County's tuberculosis screening program and it comprises 138 chest radiographs with either a resolution of 4020 × 4892 or 4892 × 4020. The Shenzhen data set was built by Shenzhen No. 3 Hospital and it consists of 566 chest radiographs with varying resolutions around 3000 × 3000. Both MC and Shenzhen datasets were annotated with lung area masks by researchers from the U.S. National Institutes of Health [57]. We resized all chest radiographs from the three datasets and their corresponding lung area annotations into the resolution of 224 × 224 to comply with most pre‐trained backbones [64, 74]. Then we employed the proportion of 70/20/10 for the split of training (173 JSRT chest radiographs, 396 Shenzhen chest radiographs, 97 MC chest radiographs), validation (49 JSRT chest radiographs, 113 Shenzhen chest radiographs, 28 MC chest radiographs), and test data set (25 JSRT chest radiographs, 57 Shenzhen chest radiographs, 13 MC chest radiographs). We included more chest radiographs in the validation data set than the test data set per the auxiliary task to develop a superior capstone for downstream lung + space segmentation.

##### Pneumothorax segmentation data set

With the auxiliary lung segmenter built on the external datasets, we trained the lung + space discriminator, the baseline pneumothorax segmenter, and the constrained pneumothorax segmenter on the Society for Imaging Informatics in Medicine (SIIM)‐American College of Radiology (ACR) Pneumothorax Segmentation data set (SIIM‐ACR) [72]. As a subset of the ChestX‐ray14 data set [75], the SIIM‐ACR data set was prepared by radiologists from SIIM and the Society of Thoracic Radiology. It comprises 2391 pneumothorax‐positive chest radiographs and matching lesion annotations with a resolution of 1024 × 1024. All chest radiographs and lesion annotations were resized into 224 × 224 and further split into training (1674 chest radiographs), validation (239 chest radiographs), and test data set (478 chest radiographs) using the ratio of 70/10/20 to involve more samples in the test set and ensure the robustness of evaluation.

#### 2.4.2 Baseline segmentation

Motivated by the previous study demonstrating that classic architectures effectively disentangle the impacts of training strategies [76], we employed six segmentation networks using three well‐established architectures of U‐Net [38], LinkNet [77], or PSPNet [78], along with two backbones of VGG‐11 [21] or MobileOne‐S0 [74] to serve as the baseline. Due to fixed input formats that preclude additional constraints and limited computational resources, automated machine learning frameworks like nnU‐Net [79] were not implemented. Furthermore, models that require human involvement, such as the segmentation anything model [80], were excluded because of the lack of necessary interaction data, specifically segmentation prompts. The model input was a grayscale chest radiograph, replicated three times on the channel level to align with most backbones. The model output was a standard single‐channel probability map with a resolution of 224 × 224, which was binarized using a common threshold of 0.5 to delineate the specific disease region [81]. The standard Dice loss was used in model training [69].

#### 2.4.3 Anatomical constraint learning

To learn well‐behaved constraints of lung + space without additional annotation in the target data set, we followed a multi‐step approach: First, we extracted constraints from an auxiliary task involving lung area segmentation using three external datasets; Next, we applied three morphological operations to transform the lung area into the lung + space. Finally, we trained a lung + space discriminator to filter out noisy constraints.

The lung segmenter in the first step was constructed based on the U‐Net architecture with the VGG‐11 backbone. The three morphological operations in the second step consisted of the following: the top two largest connected component cutoffs, closing with a 19 × 19 ellipse element [61] to fill voids within the lung area, and dilation with a 15 × 15 ellipse element [61] to smooth the lung area boundary and encompass the lung + space outside the lung area. The lung + space discriminator in the third step was based on the VGG‐11 backbone, and we adjusted its output to produce a single predictive value. The coverage rate τ in Equation (1) was set as 0.99 to generate the classification label $B_{i}$. A further step for noise filtering was to increase the binarization cutoff value to include the constraints with high confidence. We chose the cutoff value based on several high specificity values of 0.80, 0.85, 0.90, and 0.95 and subsequently optimized it as a hyper‐parameter in the constrained segmentation training.

#### 2.4.4 Constrained segmentation

After the selection via the lung + space discriminator, the well‐behaved constraints were kept, and for those samples without constraints, all‐one matrixes without penalty effect were used to ensure the data alignment in the training process. We modified the loss function in model training by adding a penalty term of Equation (4) to the standard Dice loss. The penalty term compared each pneumothorax prediction with its corresponding constraint. Hyper‐parameter λ in the modified loss function of Equation (3) was grid selected from 0.2, 0.4, 0.6, 0.8, and 1.0 according to model performance on the validation set. Despite the modification of the loss function, all other settings remained unchanged as the baseline models, including the model inputs, outputs, architectures, backbones, and optimizer, to facilitate a fair comparison.

#### 2.4.5 Implementation details

We utilized the standard Dice loss to train the lung area segmenter and the baseline pneumothorax segmenter. For the training of the lung + space discriminator, we employed the classic cross‐entropy loss. For the constrained training of the pneumothorax segmenter, we added a penalty term to the standard Dice loss. Across all experiments, stochastic gradient descent (SGD) was employed to minimize the respective loss functions. We initiated the learning rate at 0.01 and reduced it to 0.9 of its current value if no improvement was observed for five epochs on the validation data set. Table 2 provides an overview of the experimental settings.

**Table 2:** Default experimental settings in constrained segmentation.

Phase	Hyper‐parameter	Candidate
Auxiliary lung segmentation	Architecture	U‐Net
	Backbone	VGG‐11
	Loss function	Dice loss
	Optimizer	SGD
Lung + space segmentation	Closing element size	19 × 19
	Dilation element size	15 × 15
Lung + space discrimination	Cover rate	0.99
	Backbone	VGG‐11
	Loss function	Cross‐entropy
	Optimizer	SGD
Pneumothorax segmentation	Architecture	U‐Net, LinkNet, PSPNet
	Backbone	VGG‐11, MobileOne‐S0
	Loss function	Dice loss
	Optimizer	SGD

For reproducibility, the pipeline was developed in PyTorch 1.12.1, and the code has been made open access [82]. We implemented the experiments on a Dell Precision 7920 Tower Workstation with an Intel Xeon Silver 4210 CPU and an NVIDIA GeForce RTX 2080 Super GPU.

#### 2.4.6 Evaluation metrics

##### Evaluation of constraints plausibility

The anatomical constraints of lung + space, generated by the external lung segmenter and morphological operations, contained high uncertainty. To filter out the noisy constraints, we trained a lung + space discriminator and used the area under the receiver operating characteristic curve (AUROC) to assess the model performance. We set the classification thresholds according to the specificity values of 0.80, 0.85, 0.90, and 0.95 [83] and reported the sensitivity, positive predictive value (PPV), and negative predictive value (NPV) to present the characteristics of predicted constraints after selection. Besides the evaluation metrics, we also reported their respective confidence intervals via bootstrapping.

##### Evaluation of segmentation performance

We utilized the intersection over union (IoU) [84], dice similarity coefficient (DSC) [85], and the Hausdorff distance (HD) [86] to assess the pixel‐level difference between the predicted area and the ground‐truth annotation in both the auxiliary task of lung area segmentation and the target task of pneumothorax segmentation. IoU and DSC are designed to quantify the degree of overlap between the predicted area and the ground‐truth annotation. Accordingly, elevated values in these two metrics indicate enhanced model performance. On the other hand, HD assesses the distance between the two aforementioned regions, and therefore, diminished HD stands for better performance. In addition to the mean values on the test data set, we also provided the confidence intervals based on bootstrapping.

## 3 RESULTS

This section presents both quantitative and qualitative results of each module in the proposed constrained segmentation. For the quantitative evaluations, we reported the results of auxiliary lung segmentation, lung + space discrimination, and pneumothorax segmentation. An ablation study was presented to underscore the efficacy of each designed element. We further highlighted the robustness study to validate the stability of our constraint‐based formulation. For the qualitative assessments, we first showed the visual samples of lung segmentation derived from the external lung segmentation data set. We then presented the various phases involved in lung + space generation. Lastly, we compared the pneumothorax segmenter trained by using baseline and that trained using our constrained loss function.

### 3.1 Auxiliary lung area segmentation

**Figure 2:**
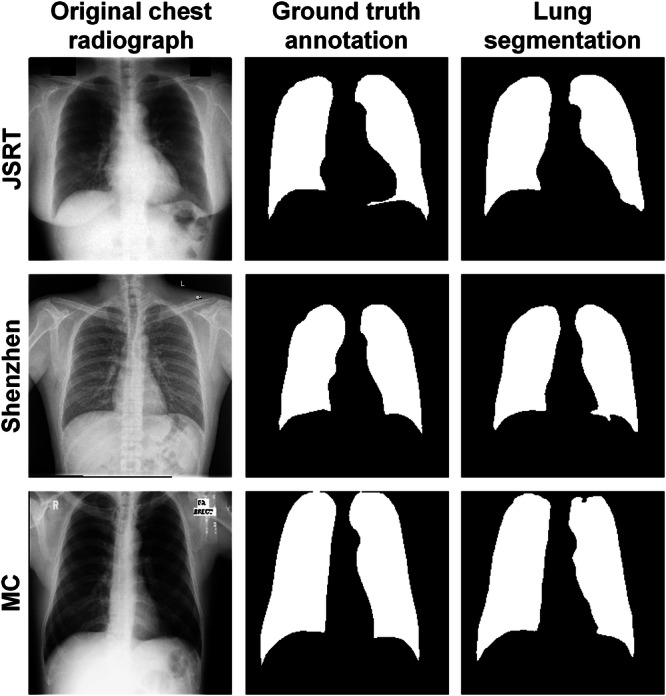
Visual samples of lung area segmentation in JSRT, Shenzhen, and MC databases.

Table 3 shows the auxiliary lung segmentation results using the U‐Net architecture and the VGG‐11 backbones. The segmenter yielded the best IoU, DSC, and HD on the test data set of the MC database and performed comparably well on the other two databases. Figure 2 visualizes three random samples and their segmentation results, demonstrating the outstanding performance of the auxiliary lung area segmenter on the external datasets.

**Table 3:** Performance of the lung segmenter on the external lung segmentation test datasets of JSRT, Shenzhen, and MC, in terms of IoU, DSC, and HD.

Datasets	IoU	DSC	HD
JSRT	0.949 (0.943–0.955)	0.974 (0.972–0.976)	3.814 (3.536–4.092)
Shenzhen	0.918 (0.902–0.934)	0.956 (0.946–0.966)	4.158 (3.807–4.509)
MC	0.961 (0.955–0.967)	0.980 (0.976–0.984)	3.732 (3.432–4.032)

*Note*: The mean value of each measurement is presented alongside its corresponding confidence interval, enclosed within brackets. Higher DSC or IoU values indicate better performance, while lower HD values demonstrate better performance.

Abbreviations: DSC, dice similarity coefficient; HD, Hausdorff distance; IoU, intersection over union.

### 3.2 Lung + space discrimination

To obtain the lung + space, we first applied the auxiliary lung segmenter on the target data set of pneumothorax segmentation to obtain the initial lung area and then processed the lung area with three morphological operations. The first operation selected the top two largest connected components, thereby eliminating noisy segment islands [87]. The second operation entailed a closing operation using an element size of 19 × 19 to fill voids within the lung area. The final operation consisted of dilation with a 15 × 15 element size to encompass the pleural space situated between the lungs and chest wall.

**Figure 3:**
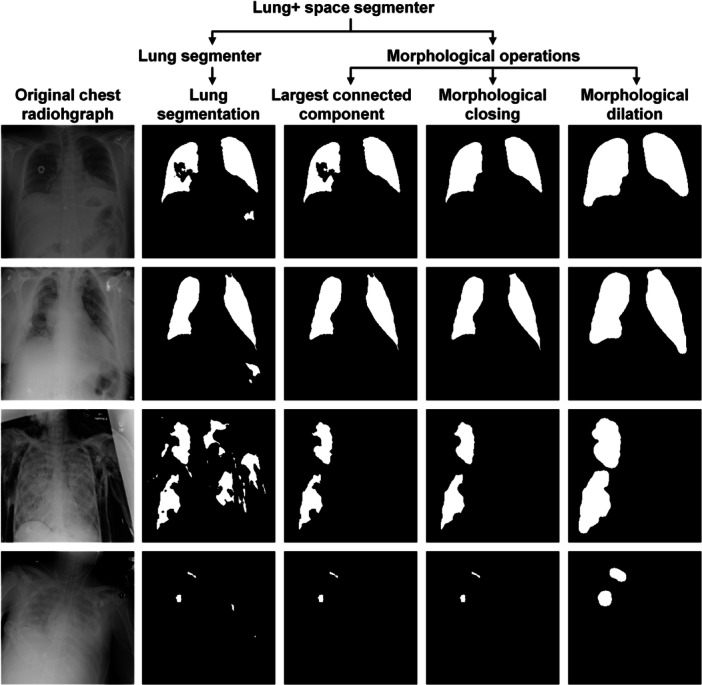
Visualization of different generation stages of the constraints of lung + space in SIIM‐ACR database.

Figure 3 presents different stages of lung segmentation and morphological operations on the SIIM‐ACR data set. Chest radiographs in the first two rows demonstrate the noisy small islands and the holes in the lung structure, which is alleviated by the morphological operations. However, it is worth noting that not all constraints can be adjusted, as depicted in the last two rows, which showcase collapsed scenarios and validate the essential role of the reliability discriminator in identifying and discarding erroneous constraints.

Based on the predicted lung + space, we compared it to the ground‐truth pneumothorax annotation. A lung + space was labeled well‐behaved if it covered 0.99 of its corresponding pneumothorax areas. With these binary labels, a discriminator was trained and tested. To further mitigate the introduction of noisy constraints in downstream training, we established cutoff values based on multiple specificity thresholds. Table 4 presents the discriminator's performance in terms of AUROC, specificity‐based cutoff values, the respective specificity, sensitivity, PPV, and NPV values.

**Table 4:** Classification results of the lung + space segmentation discriminator on the test data set of the SIIM‐ACR data set.

Cutoff value^a^	AUROC	Specificity	Sensitivity	PPV	NPV
0.68	0.750 (0.705–0.795)	0.854 (0.793–0.915)	0.394 (0.333–0.455)	0.846 (0.787–0.905)	0.410 (0.353–0.467)
0.70		0.924 (0.875–0.973)	0.244 (0.193–0.295)	0.867 (0.787–0.947)	0.376 (0.323–0.429)
0.70		0.924 (0.875–0.973)	0.244 (0.193–0.295)	0.867 (0.787–0.947)	0.376 (0.323–0.429)
0.72		0.962 (0.935–0.989)	0.147 (0.114–0.180)	0.887 (0.813–0.961)	0.358 (0.309–0.407)

*Note*: The mean value of each metric is presented alongside its corresponding confidence interval, enclosed within brackets.

Abbreviations: NPV, negative predictive value; PPV, positive predictive value.

^a^ Cutoff value was incrementally determined with a step of 0.1, utilizing specificity thresholds of 0.80, 0.85, 0.90, and 0.95 on the validation set. In our experiments, as cutoff values were escalated, shifts in specificity values were observed on the validation set, resulting in identical cutoff values across distinct specificity thresholds of 0.85 and 0.90.

### 3.3 Pneumothorax segmentation

Table 5 quantitatively compares the constrained and the baseline segmentation performance across different combinations of architectures and backbones. The constrained version consistently outperformed the baseline method and yielded average performance gains of 4.6%, 3.6%, and 3.3% in terms of IoU, DSC, and HD. Notably, our strategy achieved statistically significant improvements at the 0.05 level for HD on U‐Net and PSPNet architectures with VGG‐11 backbone, as evidenced by the nonoverlapping 95% confidence intervals shown in Table 5 [88]. While nonoverlapping confidence intervals were not observed in other scenarios, the constrained training strategy consistently improved mean values of IoU, DSC, and HD across all architectures and backbones. Moreover, U‐Net architecture with VGG‐11 backbone achieved the best performance in terms of IoU and DSC across all baseline models. Furthermore, U‐Net architecture achieved better results compared with the relatively sophisticated LinkNet or PSPNet in most scenarios, demonstrating the effectiveness of U‐Net for pneumothorax segmentation.

**Table 5:** Performance comparison of the constrained and baseline segmentation with different architectures and backbones, in terms of IoU, DSC, and HD.

Architectures	Backbones	Methods	IoU	DSC	HD
U‐Net	VGG‐11	Baseline	0.316 (0.296–0.336)	0.441 (0.417–0.465)	4.799 (4.681–4.917)
		Ours	0.336 (0.316–0.356)	0.461 (0.437–0.485)	4.558 (4.454–4.662)
		Improvement	6.3%	4.5%	5.0%
	MobileOne‐S0	Baseline	0.309 (0.287–0.331)	0.431 (0.404–0.458)	4.703 (4.605–4.801)
		Ours	0.326 (0.306–0.346)	0.449 (0.427–0.471)	4.586 (4.496–4.676)
		Improvement	5.5%	4.2%	2.5%
LinkNet	VGG‐11	Baseline	0.305 (0.287–0.323)	0.426 (0.404–0.448)	4.740 (4.652–4.828)
		Ours	0.322 (0.300–0.344)	0.447 (0.422–0.472)	4.592 (4.490–4.694)
		Improvement	5.6%	4.9%	3.1%
	MobileOne‐S0	Baseline	0.302 (0.284–0.320)	0.425 (0.403–0.447)	4.839 (4.743–4.935)
		Ours	0.320 (0.300–0.340)	0.447 (0.423–0.471)	4.675 (4.589–4.761)
		Improvement	6.0%	5.2%	3.4%
PSPNet	VGG‐11	Baseline	0.302 (0.282–0.322)	0.424 (0.400–0.448)	4.866 (4.768–4.964)
		Ours	0.307 (0.289–0.325)	0.429 (0.407–0.451)	4.660 (4.558–4.762)
		Improvement	1.7%	1.2%	4.2%
	MobileOne‐S0	Baseline	0.260 (0.242–0.278)	0.377 (0.355–0.399)	5.008 (4.900–5.116)
		Ours	0.267 (0.247–0.287)	0.382 (0.358–0.406)	4.935 (4.831–5.039)
		Improvement	2.7%	1.3%	1.5%
Average improvement (%)			4.6	3.6	3.3

*Note*: The mean value of each measurement is presented alongside its corresponding confidence interval, enclosed within brackets. Higher DSC or IoU values indicate better performance, while lower HD values demonstrate better performance.

Abbreviations: DSC, dice similarity coefficient; HD, Hausdorff distance; IoU, intersection over union.

**Figure 4:**
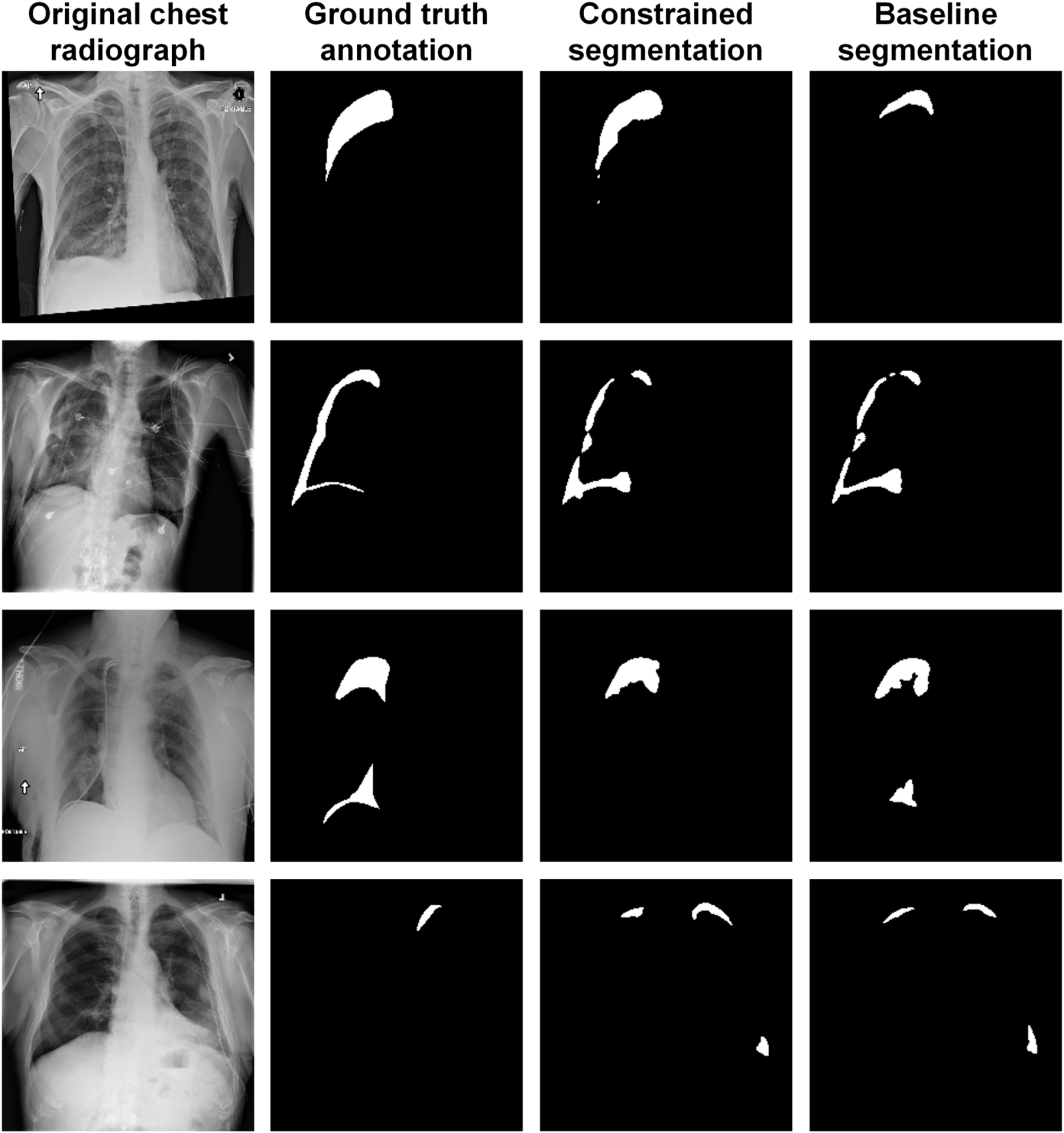
Comparative example of the constrained segmentation and the baseline segmentation. Rows 1–4 represent the following scenarios: the constrained method outperforming the baseline, the constrained method achieving comparable results to the baseline, the constrained method underperforming the baseline, and both methods collapsing, respectively.

Figure 4 provides a comparative visualization of pneumothorax segmentation between the constrained and baseline segmenters using the architecture of U‐Net and the backbone of VGG‐11. The first row exemplifies a scenario where the proposed method surpasses the baseline model, the second row presents a scenario where the proposed method achieves performance on par with the baseline model, and the third row outlines a situation where the proposed method underperformed the baseline model. The last row illustrates cases where both methods encounter difficulties.

### 3.4 Ablation study

To ascertain the efficacy of each component within the proposed formulation, we conducted an ablation study on the U‐Net architecture and the VGG‐11 backbone. As shown in Table 6, the constraints generated by the external lung area segmenter exhibited excessive noise, resulting in subpar performance compared to the baseline without any constraints. While training with constraints derived from the lung + space segmenter yielded better performance, it only achieved performance marginally better than the baseline. In contrast, with the integration of a lung + space discriminator, the noisy constraints were efficiently filtered, preserving the well‐behaved ones, and leading to substantial improvements across all evaluation metrics.

**Table 6:** Ablation study on the lung + space segmentation model and the lung + space segmentation discriminator.

Methods	Lung area segmenter	Lung + space segmenter	Lung + space discriminator	IoU	DSC	HD
Baseline	×	×	×	0.316 (0.296–0.336)	0.441 (0.417–0.465)	4.799 (4.681–4.917)
	√	×	×	0.298 (0.278–0.318)	0.421 (0.397–0.445)	4.895 (4.791–4.999)
	×	√	×	0.317 (0.297–0.337)	0.439 (0.415–0.463)	4.770 (4.682–4.858)
Ours	×	√	√	0.336 (0.316–0.356)	0.461 (0.437–0.485)	4.558 (4.454–4.662)

Abbreviations: DSC, dice similarity coefficient; HD, Hausdorff distance; IoU, intersection over union.

### 3.5 Robustness to the constraint settings

Finally, we investigated the robustness of hyper‐parameters in anatomical constraint learning. To this end, we validated a range of values in the morphological sizes and cover rates. In each validation, the architecture was configured as U‐Net, employing VGG‐11 as the specific backbone, while all other parameters were maintained at their default values, as outlined in Table 2. Tables 7 and 8 detail the ablation results of various morphological sizes and cover rates. As anticipated, the constrained model consistently exhibited superior performance than the baseline model across varying closing element sizes, dilation element sizes, and coverage rates. Notably, the closing element size of 25 × 25 and the dilation element size of 20 × 20 in Table 7 attained a new state‐of‐the‐art performance, surpassing the default parameter settings [61]. Similarly, the cover rate of 0.90 in Table 8 presented better performance than the default setting of 0.99. These observations suggest that our proposed constrained training could be further enhanced through hyper‐parameter optimization.

**Table 7:** Robustness study on morphological element sizes in the lung + space segmenter.

Methods	Closing element size	Dilation element size	IoU	DSC	HD
Baseline	×	×	0.316 (0.296–0.336)	0.441 (0.417–0.465)	4.799 (4.681–4.917)
Ours	15 × 15	10 × 10	0.331 (0.311–0.351)	0.458 (0.434–0.482)	4.691 (4.603–4.779)
		15 × 15	0.338 (0.316–0.360)	0.462 (0.438–0.486)	4.439 (4.343–4.535)
		20 × 20	0.338 (0.318–0.358)	0.463 (0.439–0.487)	4.538 (4.438–4.638)
	19 × 19^a^	10 × 10	0.332 (0.312–0.352)	0.455 (0.431–0.479)	4.521 (4.435–4.607)
		15 × 15^a^	0.336 (0.316–0.356)	0.461 (0.437–0.485)	4.558 (4.454–4.662)
		20 × 20	0.340 (0.320–0.360)	0.465 (0.441–0.489)	4.488 (4.374–4.602)
	25 × 25	10 × 10	0.339 (0.317–0.361)	0.463 (0.436–0.490)	4.442 (4.364–4.520)
		15 × 15	0.337 (0.319–0.355)	0.465 (0.441–0.489)	4.570 (4.482–4.658)
		20 × 20	0.346 (0.324–0.368)	0.473 (0.448–0.498)	4.372 (4.280–4.464)

Abbreviations: DSC, dice similarity coefficient; HD, Hausdorff distance; IoU, intersection over union.

^a^ Parameter settings in the prior literature [61].

**Table 8:** Robustness study on cover rates in the lung + space discriminator.

Methods	Cover rate	IoU	DSC	HD
Baseline	×	0.316 (0.296–0.336)	0.441 (0.417–0.465)	4.799 (4.681–4.917)
Ours	0.80	0.334 (0.314–0.354)	0.460 (0.436–0.484)	4.408 (4.306–4.510)
	0.90	0.343 (0.321–0.365)	0.470 (0.445–0.495)	4.474 (4.380–4.568)
	0.99	0.336 (0.316–0.356)	0.461 (0.437–0.485)	4.558 (4.454–4.662)

Abbreviations: DSC, dice similarity coefficient; HD, Hausdorff distance; IoU, intersection over union.

## 4 DISCUSSION

Conventional DL‐based lesion segmentation models typically employ an end‐to‐end approach, directly mapping input medical images into lesion delineations without accounting for clinical knowledge, such as the spatial distribution of the disease. In this study, we introduced a loss function that incorporates disease occurrence area as a constraint into the standard training framework. Through numerical studies, we demonstrated the effectiveness of the proposed training strategy on pneumothorax segmentation, a condition sensitive to location, manifesting in the pleural space between the lungs and the chest wall. Consistently, our proposed training method outperformed the baseline approach across three different architectures paired with two encoders. Moreover, our proposed method can be seamlessly integrated with the existing segmentation studies focusing on model architectures or training strategies. For instance, Wang et al. developed an ensemble approach integrating two architectures with four encoders based on Dice loss [40]. Abedalla et al. introduced a two‐stage method for pneumothorax segmentation: first training a U‐Net on low‐resolution chest radiographs, then fine‐tuning it with high‐resolution images using a combination of Dice and cross‐entropy loss [39]. These solutions may benefit from adding our proposed penalty term to their original loss functions [89].

While our method was showcased for pneumothorax segmentation, its applicability extends to other thoracic diseases with location‐relevant characteristics [90]. Many of these conditions exhibit location‐sensitive characteristics within the lung area [91, 92]. For thoracopathy localized in the lung area, removing the morphological dilation and utilizing the resulting lung area can potentially improve the model performance using our proposed constrained training strategy. Additionally, the proposed method can be adapted to thoracopathy localization tasks that require a bounding box around the lesion area pre‐specified by experts. A two‐step method would be to first generate pixel‐level constraints of lung area and identify the rectangular boxes encompassing these constraints, and then to integrate the penalty term in Equation (3) into the classic L2 distance‐based loss function, ensuring the model's output remains within the bounding box‐based constraints.

In contrast to the previous constraint learning methods [53, 67] that required additional annotation on the target data set and task, our method employs a four‐phase pipeline to generate sample‐specific constraints. This sets a valuable benchmark for the development of constrained models in various domains. Our method can be generalized by (1) generating initial constraints based on an auxiliary task and external datasets, (2) refining these initial constraints based on the relationship between the auxiliary and the target tasks, and (3) employing the target data set and available annotations to filter out noisy constraints, retaining only the informative ones. As highlighted in Table 6, our ablation experiment indicates that the initial constraints transferred from external datasets can introduce detrimental noise, potentially hindering model convergence. However, after refining and selecting constraints, the model's performance improves. Tables 7 and 8 further demonstrate the robustness of our method to hyper‐parameter variations in the second and third stages of learning constraints. In fact, segmenters trained under various settings outperformed the ones under the default setting, suggesting a promising avenue for future hyper‐parameter optimization.

While the proposed method has demonstrated consistent and robust improvements, there are several limitations to be addressed in future work. First, our model involves multiple hyper‐parameters. Although we have made initial adjustments, there is potential for further optimization. Intuitively, we hypothesize that there exists a certain relationship between the hyper‐parameters [79]. For instance, extensive morphological operations can broaden constraints' boundaries, suggesting a need to adjust the cover rate to mitigate noise from these expanded boundaries [93]. Second, while this work primarily incorporates anatomical shapes as constraints, future research will delve into other geometric attributes such as sphericity, convexity, and roundness [94, 95]. It is of interest to explore their impact on pneumothorax segmentation. Third, the present study was restricted by the limited computational resources, the lack of segmentation prompts, the restricted access to commercial software, the absence of imaging conditions and diverse modalities, and the vacancy of patient demographics, hindering the implementation of automated machine learning frameworks such as nnU‐Net [79], interactive models such as segmentation anything model [80], commercial segmentation solutions such as Siemens AI‐Rad Companion [96, 97], robustness studies on imaging conditions and diverse modalities, and subpopulation evaluations [98]. Future research will collaborate with ML engineers and medical experts to launch these experiments and explore how trained physicians can benefit from these DL tools [44, 99]. Lastly, previous studies demonstrate a strong correlation between anatomical information and the diagnosis of various thoracic diseases [62, 100]. Therefore, we plan to evaluate the adaptability of the proposed method across a broader spectrum of thoracopathy tasks, including classification, detection, and segmentation [101, 102].

## 5 CONCLUSIONS

Historically, domain knowledge was underemphasized by the DL community when tackling medical tasks. In this study, we underscore the value of integrating clinical knowledge, particularly regarding disease occurrence, to enhance DL‐based pneumothorax segmentation. Different from previous work that requires additional annotation on the target data set and task, our approach leverages external open‐access datasets and an auxiliary task. This strategy not only streamlines our process but also offers versatility, making it a promising framework for diagnosing other thoracic conditions or diseases with location‐relevant characteristics.

## AUTHOR CONTRIBUTIONS

**Han Yuan**: Conceptualization (equal); data curation (equal); formal analysis (equal); methodology (equal); software (equal); writing—original draft (equal); writing—review and editing (equal). **Chuan Hong**: Conceptualization (equal); formal analysis (equal); investigation (equal); methodology (equal); validation (equal); writing—review and editing (equal). **Nguyen Tuan Anh Tran**: Investigation (equal); validation (equal); writing—review and editing (equal). **Xinxing Xu**: Investigation (equal); validation (equal); writing—review and editing (equal). **Nan Liu**: Conceptualization (equal); funding acquisition (equal); investigation (equal); methodology (equal); project administration (equal); resources (equal); supervision (equal); writing—review and editing (equal).

## CONFLICT OF INTEREST STATEMENT

The authors declare no conflict of interest.

## ETHICS STATEMENT

Ethics approval was not required for this study as it utilized retrospective datasets that are publicly accessible. Researchers seeking access to the original data should request permission from the data owners and comply with their established protocols on data privacy and confidentiality.

## INFORMED CONSENT

Not applicable.

## Data Availability

Data sharing is not applicable to this article as no data sets were generated or analyzed during the current study.
